# Nano-Welding of Multi-Walled Carbon Nanotubes on Silicon and Silica Surface by Laser Irradiation

**DOI:** 10.3390/nano6030036

**Published:** 2016-02-24

**Authors:** Yanping Yuan, Jimin Chen

**Affiliations:** 1 Institute of Laser Engineering, Beijing University of Technology, Beijing 100124, China; jimin@bjut.edu.cn; 2 Beijing Engineering Research Center of 3D Printing for Digital Medical Health, Beijing University of Technology, Beijing 100124, China

**Keywords:** nano-welding, multi-walled carbon nanotubes, laser

## Abstract

In this study, a continuous fiber laser (1064 nm wavelength, 30 W/cm^2^) is used to irradiate multi-walled carbon nanotubes (MWCNTs) on different substrate surfaces. Effects of substrates on nano-welding of MWCNTs are investigated by scanning electron microscope (SEM). For MWCNTs on silica, after 3 s irradiation, nanoscale welding with good quality can be achieved due to breaking C–C bonds and formation of new graphene layers. While welding junctions can be formed until 10 s for the MWCNTs on silicon, the difference of irradiation time to achieve welding is attributed to the difference of thermal conductivity for silica and silicon. As the irradiation time is prolonged up to 12.5 s, most of the MWCNTs are welded to a silicon substrate, which leads to their frameworks of tube walls on the silicon surface. This is because the accumulation of absorbed energy makes the temperature rise. Then chemical reactions among silicon, carbon and nitrogen occur. New chemical bonds of Si–N and Si–C achieve the welding between the MWCNTs and silicon. Vibration modes of Si_3_N_4_ appear at peaks of 363 cm^−1^ and 663 cm^−1^. There are vibration modes of SiC at peaks of 618 cm^−1^, 779 cm^−1^ and 973 cm^−1^. The experimental observation proves chemical reactions and the formation of Si_3_N_4_ and SiC by laser irradiation.

## 1. Introduction

The discovery of carbon nanotubes (CNTs) in 1991 [1] has attracted tremendous attention. Due to their superior electrical, mechanical, and thermal properties [2,3], CNTs are unique building blocks for many novel functional materials, which gives CNTs enormous commercial potential in applications including energy storage [4] and biotechnology [5]. In order to expand their applications, it is necessary to achieve nanoscopic welding which is one of the promising ways to rearrange, assemble and integrate CNTs for nanoscale systems and nanodevices [6]. In the past decade, some approaches have been proposed, including mechanical manipulation with the atomic force microscope [7], electron beam irradiation [8], ion irradiation [9], heat welding [10,11,12,13] and welding by laser irradiation [14,15,16,17]. However, the nanoscale welding, including CNTs-CNTs and CNTs-substrates, is still under discussion, and requires more investigation to achieve reliable junctions.

In this study, a continuous fiber laser is used to irradiate MWCNTs on different substrate (silicon and silica) surfaces. The effects of substrates on the welding of MWCNTs are studied. We investigate the joining of MWCNTs and the welding between MWCNTs and silicon. The experiments demonstrate that: (1) substrates strongly affect the welding process of MWCNTs; (2) more irradiation time is needed to weld MWCNTs on a silicon surface due its higher thermal conductivity; (3) by prolonging laser irradiation time, the chemical reaction products Si_3_N_4_ and SiC are created during the welding processing between MWCNTs and silicon. New chemical bonds of Si–N and Si–C make the welding between MWCNTs and silicon successful.

## 2. Results and Discussion

**Figure 1:**
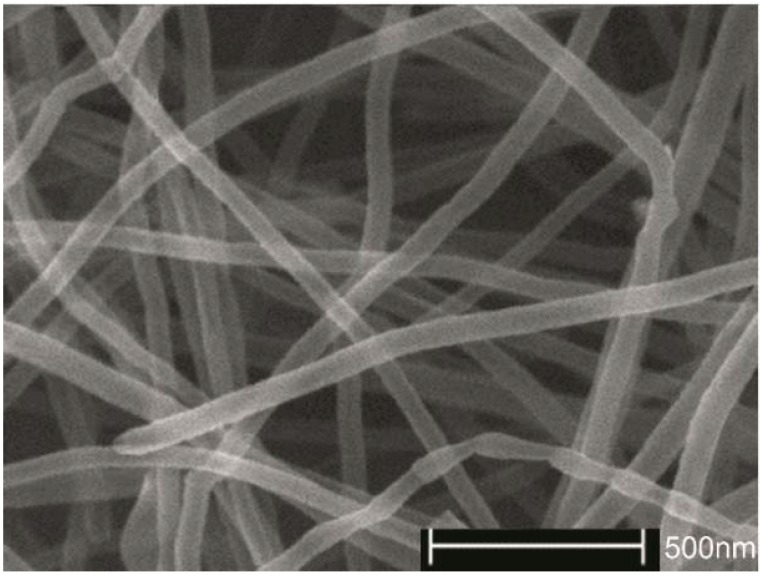
scanning electron microscope (SEM) image of raw multi-walled carbon nanotube (MWCNT) samples.

The SEM image of a raw MWCNT sample is shown in Figure 1. Smooth, straight and uniform tube walls of MWCNTs are observed, which almost have no structural defects. MWCNTs have high aspect (length/diameter) ratios.

### 2.1. Effects of Substrates

**Figure 2:**
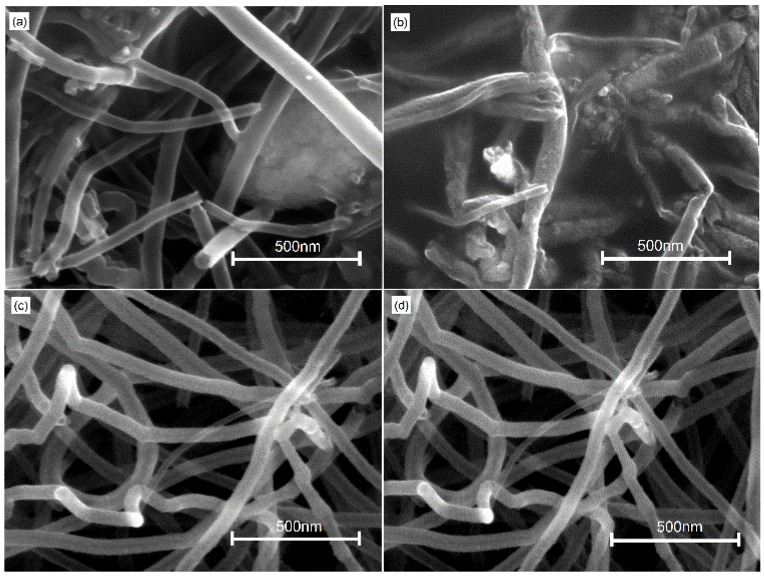
SEM images of crossed MWCNTs after 1064 nm laser irradiation on different substrates: (**a**) 3 s, silica; (**b**) 6 s, silica; (**c**) 6 s, silicon; and (**d**) 8 s, silicon.

Figure 2 shows SEM images of crossed MWCNTs after 1064 nm laser irradiation on the substrates of silica and silicon: (a) 3 s irradiation on silica; (b) 6 s irradiation on silica; (c) 6 s irradiation on silicon; and (d) 8 s irradiation on silicon. As shown in Figure 2a, after 3 s irradiation, nanoscale welded joints with good quality are created due to the absorption of laser energy. The energy is absorbed by electrons of MWCNTs and then transformed to the atoms of MWCNTs. The collision of phonon with a carbon atom results in the formation of radiation defects such as vacancies and interstitials in the walls of MWCNTs. Due to the moderate temperature, the mobility of irradiation defects leads to C–C bond breaks in various layers of MWCNTs [18]. Then new chemical bonds are formed in the contact interface of both welding carbon tubes, which leads to the formation of new graphene layers. New graphene layers between crossed nanotubes make the nanoscale welding successful. When the irradiation time is prolonged up to 6 s, the morphology of MWCNTs changes a lot, and structures with lower aspect ratio are formed. Crossed MWCNTs are still welded together, as shown in Figure 2b, because pairs of heptagons, pentagons and holes are formed [19].

As compared with the cases shown in Figure 2a,b, after 6 or 8 s irradiation, MWCNTs on the surface of silicon substrate appear the same and are not welded yet. One observation is the tendency of MWCNTs to bend and curve. The difference of MWCNTs on silica and silicon substrates is attributed to various thermal conductivities. The thermal conductivity of silicon is about 140.0 W·m^−1^·K^−1^ [20], while the coefficient of silica is about 1.0 W·m^−1^·K^−1^ [21]. Due to the higher thermal conductivity of silicon, it is not easy to accumulate the absorbed energy on the surface of MWCNTs. Hence, more irradiation time is needed for the nanoscale welding of MWCNTs on silicon.

### 2.2. Nanowelding between Carbon Nanotubes

**Figure 3:**
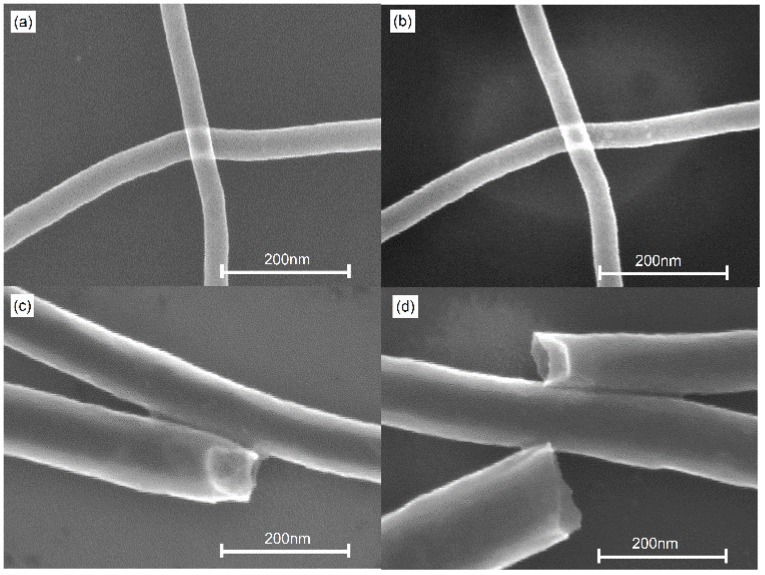
SEM images of (**a**) raw MWCNT samples and (**b**) crossed MWCNTs after 10 s laser irradiation, (**c**,**d**) parallel MWCNTs after 10 s laser irradiation.

In order to investigate the welding of MWCNTs on the silicon substrate, the irradiation time is prolonged up to 10 s. Figure 3 shows SEM images of Figure 3a raw MWCNTs; Figure 3b crossed MWCNTs after 10 s laser irradiation; Figure 3c,d parallel MWCNTs after 10 s laser irradiation. The only difference between Figure 3a and Figure 3b is the brightness change, which is hard to observe the quality of welding. Hence, we focus on the investigation of parallel MWCNTs. From Figure 3c,d, it is found that the joints are created by laser irradiation. The nanoscale welded joint (as shown in Figure 3c,d is similar to the welding case after 3 s laser irradiation on silica. Hence, the nanoscale welding is attributed to the absorption of laser energy. The absorbed energy leads to C–C bonds breaking in various layers of MWCNTs. The formation of new chemical bonds leads to the formation of new graphene layers between crossed nanotubes, which makes the nanoscale welding successful.

### 2.3. Nanowelding between Carbon Nanotubes and Silicon

**Figure 4:**
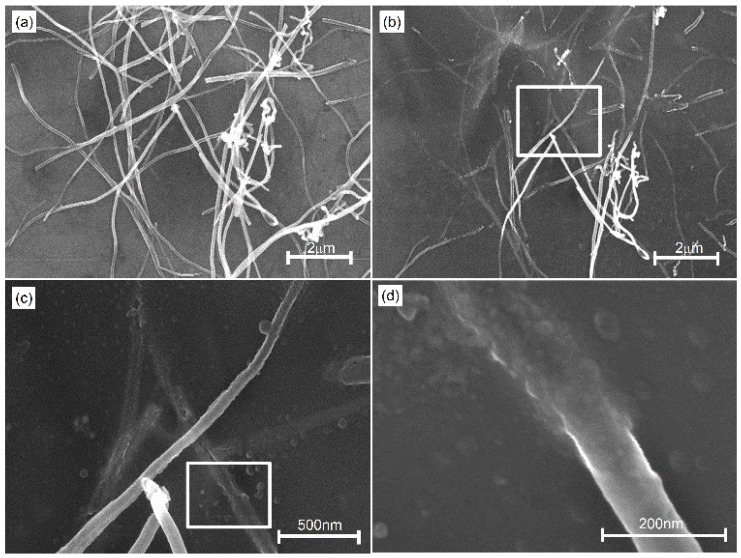
SEM images of MWCNTs samples: (**a**) raw MWCNTs on silicon surface; (**b**) MWCNTs after 12.5 s laser irradiation; (**c**) the magnified SEM image of white rectangle in Figure 4b; and (**d**) the magnified SEM image of white rectangle in Figure 4c.

In this study, the welding between MWCNTs and the silicon substrate is also investigated by SEM. Figure 4a shows the SEM image of dispersed raw MWCNT samples on the silicon surface, which is evenly dispersed by 0.1% Triton X-100 (SPI Supplies, West Chester, PA, USA). The SEM image of MWCNTs after 12.5 s irradiation is shown in Figure 4b. It is found that most of the MWCNT samples on silicon welded to the substrate after laser irradiation and the frameworks of the MWCNT tube walls are visible on the silicon surface. The magnification of the white rectangle in Figure 4b is shown in Figure 4c. The frameworks of tube walls on the silicon surface are clear. Some structures with lower aspect ratios are formed on the silicon surface. The connection section between MWCNTs and silicon (white rectangle in Figure 4c) is magnified, as shown in Figure 4d. It is found that the nanoscale welding between MWCNTs and silicon can be achieved by adjusting the irradiation time, which is important for applications.

**Figure 5:**
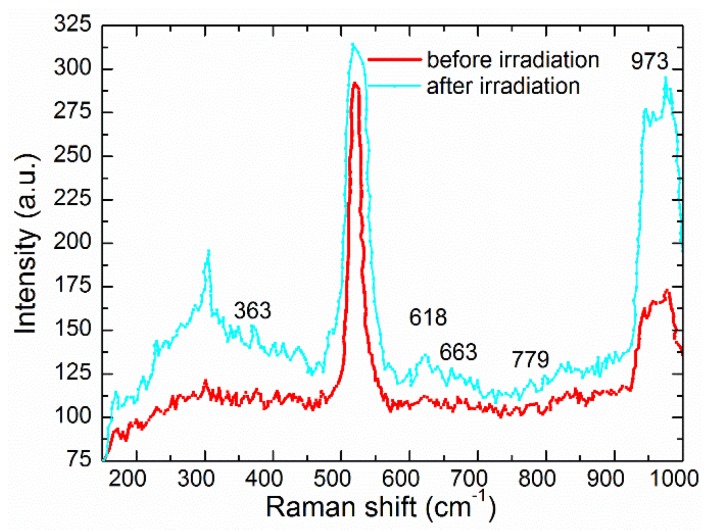
Raman spectra of MWCNTs. (**Red**) before laser irradiation; (**Blue**) after 12.5 s laser irradiation.

In this study, we also investigate the mechanisms of nanoscale welding between MWCNTs and silicon. Due to the higher thermal conductivity coefficient of silicon substrate, the absorbed energy is conducted from MWCNTs to silicon, which leads to more irradiation time being required to achieve nanoscale welding. With the prolonging of the irradiation time, the thermal effects lead to the temperature rising. At certain temperatures, a compound combination reaction occurs among the silicon (substrate), carbon nanotubes and nitrogen (shielding gas) [22,23,24]. When the temperature reaches 1200 °C, the combination reaction between silicon and nitrogen occurs and the combined product (Si_3_N_4_) is created, as shown in the chemical reaction (1). As the temperature rises up to 1400 °C, the chemical reaction between Si_3_N_4_ and C can create SiC and N_2_. With the temperature rising up to 1800 °C, the combination reaction between Si and C occurs, which creates the combined product (SiC). These chemical reactions are proved by Raman spectra. Raman spectra of MWCNTs before irradiation and after 12.5 s irradiation are shown in Figure 5. It is found that the vibration modes of Si_3_N_4_ appear at peaks of 363 cm^−1^, and 663 cm^−1^ [25]. There are vibration modes of SiC at peaks of 618 cm^−1^, 779 cm^−1^ and 973 cm^−1^ [26]. From Figure 5, it is proved that Si_3_N_4_ and SiC are created by laser irradiation. New chemical bonds of Si–N and Si–C make the welding between MWCNTs and substrates successful. The basic reactions involved in the formation of the Si_3_N_4_ and SiC are given by:

(1) $$3\text{Si}+2{\text{N}}_{2}\overset{1200{}^{\circ}\text{C}}{=}{\text{Si}}_{3}N_{4}$$

(2) $${\text{Si}}_{3}N_{4}+3\text{C}\overset{1400{}^{\circ}\text{C}}{=}3\text{SiC}+{\text{N}}_{2}$$

(3) $$\text{Si}+\text{C}\overset{1800{}^{\circ}\text{C}}{=}\text{SiC}$$

## 3. Experimental Set-Up

**Figure 6:**
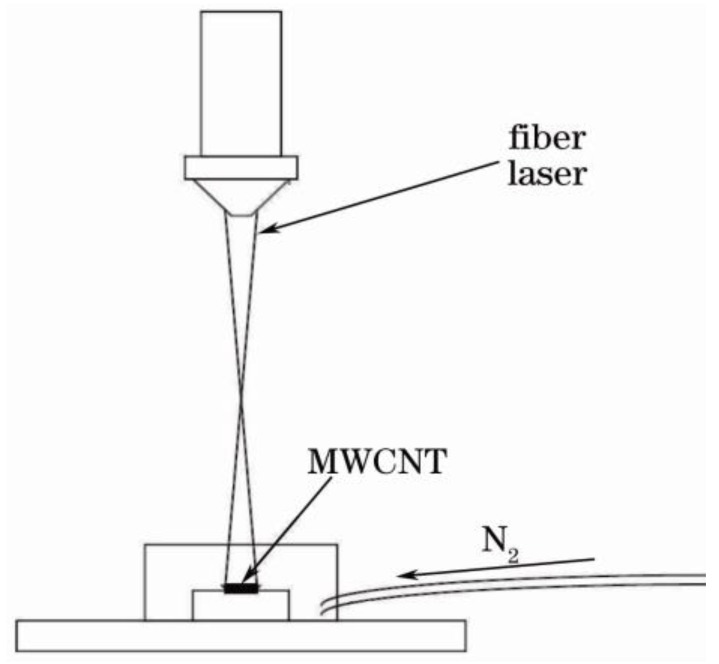
The setup of MWCNT samples irradiated by fiber laser.

A continuous fiber laser (IPG Photonics, YLR-100-SM-AC, IPG Photonics, Boston, MA, USA) is used to generate lasers in our experiments. The central wavelength is about 1064 nm. The diameter of the focused spot size is about 5 μm. The power of the laser can be adjusted with the range from 1 W to 100 W. The substrates used in our experiments are silica and silicon. MWCNTs with 60 nm–70 nm diameters and 5 μm–7 μm lengths are synthesized by the catalytic chemical vapor deposition (CCVD) method. Due to van der Waals attraction between nanotubes, MWCNTs tend to form agglomerates. The 0.1% Triton X-100 is used as a dispersing agent. The substrate samples are cleaned in an ultrasonic bath with anhydrous ethanol (Jinan Shengquan Group Share-Holding Co., Ltd., Jinan, China), acetone (Shanghai Hanhong Chemical Co., Ltd., Shanghai, China) and deionized water (Taiyuan Lanlang Technology Industrial Corp., Taiyuan, China) for 5 min and then dried by high pressure nitrogen; MWCNTs agglomerates dispersed evenly by ultrasonic are dipped on the surface of substrate samples. Then the continuous fiber laser irradiates the MWCNT sample. In our experiments, the processing power density is 30 W/cm^2^. For the MWCNTs on silica substrate, the irradiation time is 3 s and 6 s. In this study, we investigate the morphologies of MWCNTs on silicon substrate after 6 s, 8 s, 10 s and 12.5 s laser irradiation by SEM (Scanning electron microscopy) (FEI XL30 S-FEG, FEI, Hillsboro, OR, USA) is used in our experiments, and the maximum magnification is 400,000 times). Nitrogen is used as shielding gas. In order to investigate the mechanisms of nanoscale welding between MWCNTs and silicon, the prepared substrates are tested by a Raman spectrometer (HORIBA Jobin Yvon, Paris, France). The setup of MWCNTs samples irradiated by fiber laser is as shown in Figure 6.

## 4. Conclusions

In this study, a continuous fiber laser (1064 nm wavelength, 30 W/cm^2^) is used to irradiate MWCNTs on different substrate surfaces. It is found that substrates have significant effects on the welding process. For the MWCNTs on the silica substrate, due to breaking C–C bonds and new graphene layers, junctions with good quality are formed after 3 s irradiation. Carbon tube walls with some defects are observed after 6 s irradiation. For the MWCNTs on the silicon substrate, the welding junctions can be formed until 10 s. This is attributed to the difference of thermal conductivity between silica and silicon. As the irradiation time is prolonged up to 12.5 s, most of the MWCNTs just keep their frameworks of tube walls on the surface of the silicon, which makes the nano-welding between MWCNTs and silicon successful. Due to the accumulation of absorbed energy, the temperature keeps rising during the welding process, which leads to chemical reactions among the silicon, carbon and nitrogen. The chemical reactions (new chemical bonds of Si–N and Si–C) make nanoscale welding between MWCNTs and silicon successful. After the chemical reactions, the products of Si_3_N_4_ and SiC are created. Vibration modes of Si_3_N_4_ appear at peaks of 363 cm^−1^ and 663 cm^−1^ and vibration modes of SiC appear at peaks of 618 cm^−1^, 779 cm^−1^ and 973 cm^−1^, which proves the formation of Si_3_N_4_ and SiC. The welding between MWCNTs and silicon substrates is very important for nanotechnology.
